# Interactions of Spike-RBD of SARS-CoV-2 and Platelet Factor 4: New Insights in the Etiopathogenesis of Thrombosis

**DOI:** 10.3390/ijms22168562

**Published:** 2021-08-09

**Authors:** Margherita Passariello, Cinzia Vetrei, Felice Amato, Claudia De Lorenzo

**Affiliations:** 1 Ceinge—Biotecnologie Avanzate s.c. a.r.l., via Gaetano Salvatore 486, 80145 Naples, Italy; margherita.passariello@unina.it (M.P.); cinzia.vetrei@unina.it (C.V.); felice.amato@unina.it (F.A.); 2 Department of Molecular Medicine and Medical Biotechnology, University of Naples “Federico II”, Via Pansini 5, 80131 Naples, Italy

**Keywords:** SARS-CoV2, Spike-RBD, PF4, antibodies, thrombosis

## Abstract

The rare but dangerous adverse events evidenced after massive vaccination against SARS-CoV-2 are represented by thrombosis and thrombocytopenia. The patients diagnosed with severe COVID-19 may develop a pro-thrombotic state with a much higher frequency, thus we decided to investigate the role of Spike protein (the only common product of the two conditions) or the anti-Spike antibodies in the etiopathogenesis of thrombosis. A pathogenic Platelet Factor 4 (PF4)-dependent syndrome, unrelated to the use of heparin therapy, has been reported after the administration of vaccines in the patients manifesting acute thrombocytopenia and thrombosis. Thus, we aimed at shedding light on the structural similarities of Spike of SARS-CoV-2 and PF4 on their eventual biochemical interactions and on the role of their specific antibodies. The similarities between PF4 and Spike-RBD proteins were evaluated by a comparison of the structures and by testing the cross-reactivity of their specific antibodies by ELISA assays. We found that the anti-Spike antibodies do not recognize PF4, on the contrary, the anti-PF4 antibodies show some cross-reactivity for Spike-RBD. More interestingly, we report for the first time that the PF4 and Spike-RBD proteins can bind each other. These data suggest that the interaction of the two proteins could be involved in the generation of anti-PF4 antibodies, their binding to Spike-RBD, which could lead to platelets aggregation due also to their high expression of ACE2.

## 1. Introduction

The massive vaccination against SARS-CoV-2 highlighted the occurrence of rare but serious side effects not previously evidenced during the clinical trials. Some days after the initial vaccination with ChAdOx1 nCov-19 (AstraZeneca), some cases of adverse events characterized by thrombosis and thrombocytopenia [1,2,3], observed also in patients with severe COVID-19 [4,5], have been reported in healthy people with no previous prothrombotic events or hereditary thrombophilia.

The first diagnostic hypothesis of vaccine-induced immune thrombotic thrombocytopenia (VITT) was based on the observation in vaccinated patients of platelets hyperactivity, associated with a decreased overall platelet count [6], increased *D*-dimer [7]** and high levels of antibodies to Platelet Factor 4 (PF4) [8,9]. Moreover, it has been recently reported in literature that platelets express high levels of angiotensin converting enzyme 2 (ACE2), the host cell receptor for SARS-CoV-2, and the transmembrane serine protease 2 (TMPRSS2), responsible for Spike protein priming [8]. Upon activation, platelets also secrete PF4 [10,11], a small chemokine whose main physiological function is to regulate blood coagulation. 

It is well known that heparin binding to PF4 can induce the generation of anti-PF4 antibodies [8] in cases of heparin-induced thrombocytopenia (HIT) [12], hence, PF4 could exert a similar key role in the cases of VITT. Moreover, recently structural similarities have been evidenced between this chemokine secreted by activated platelets and the Spike protein expressed on SARS-CoV-2 [13].

On the basis of these evidences, we investigated the possible role of both Spike-RBD and PF4 proteins and their respective antibodies (Ab) in the etiopathogenesis of thrombosis in COVID-19 severe patients and in vaccinated people experiencing VITT. Therefore, we compared the structures of the two proteins, evaluated the cross-reactivity of their antibodies and analyzed their eventual interactions considering the common ability of both the proteins to form oligomers [14,15].

## 2. Results

### 2.1. Structural Similarity between Spike-RBD and PF4 Proteins

**Figure 1:**
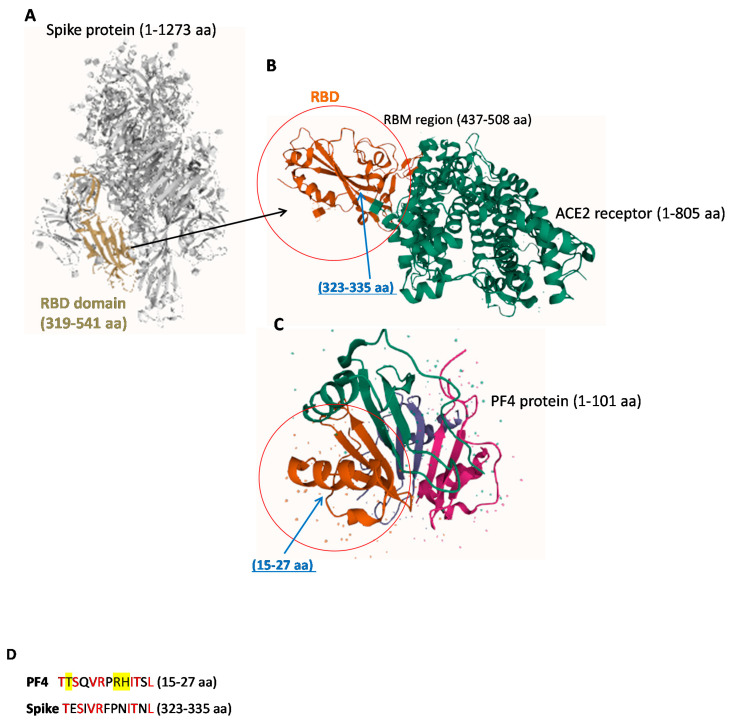
Representative images of 3D structures of SARS-CoV-2 RBD, ACE-2 and PF4 proteins. (**A**) SARS-CoV-2 spike ectodomain structure (open state, 6vyb) [18]. The RBD domain (319–541 aa) is highlighted in gold. (**B**) Crystal structure of SARS-CoV-2 Spike Receptor-Binding Domain bound with ACE2 (6vw1). (**C**) Crystal structure of platelet factor 4 1f9q. The different domains of the protein are indicated by different colors: the chemokine interleukin-8-like domain is shown by pink color, the interleukin 8-like chemokine domain in green; the CXC Chemokine domain in brown and the small cytokine C-X-C is shown in purple color [17]. In the red circles are highlighted the domains of the Spike-RBD protein (**B**) and PF4 protein (**C**) which show a similarity in the structure with the common sequences [1] indicated by blue arrows (**D**). All the structures were obtained by PDB IntrePro. The PF4 aa residues involved in VITT are highlighted in yellow (**D**). The Red color of the Figure 1D indicates identical residues in the two sequences.

It has been reported in literature that antibodies specific for Platelet Factor 4 (PF4) were present at high levels in patients who presented thrombosis after vaccination with the AstraZeneca ChAdOx1 nCoV-19 vaccine [8,16]. Since PF4 contains regions with sequence identities with Spike-RBD, as that reported in Figure 1D [1], we compared the 3D structures of the two indicated proteins to verify whether their structural similarity could provoke the cross-reactivity of antibodies specific for each protein for the other one [6]. 

We found that the two proteins show similarity in the regions including anti-parallel βsheets surrounded by two αhelices, evidenced by the circles in Figure 1B,C, containing common sequences corresponding to 323–335 amino acidic (aa) residues in RBD (Figure 1B) and to 15–27 aa residues in the CXC Chemokine domain of PF4 (Figure 1C) [1,17]. Other homologies between PF4 and Spike have been also reported either in the RBD or in the other domains of the Spike protein [13].

### 2.2. Analysis of the Cross-Reactivity of the Anti-PF4 or the Anti-Spike-RBD Antibodies for the Two Proteins

**Figure 2:**
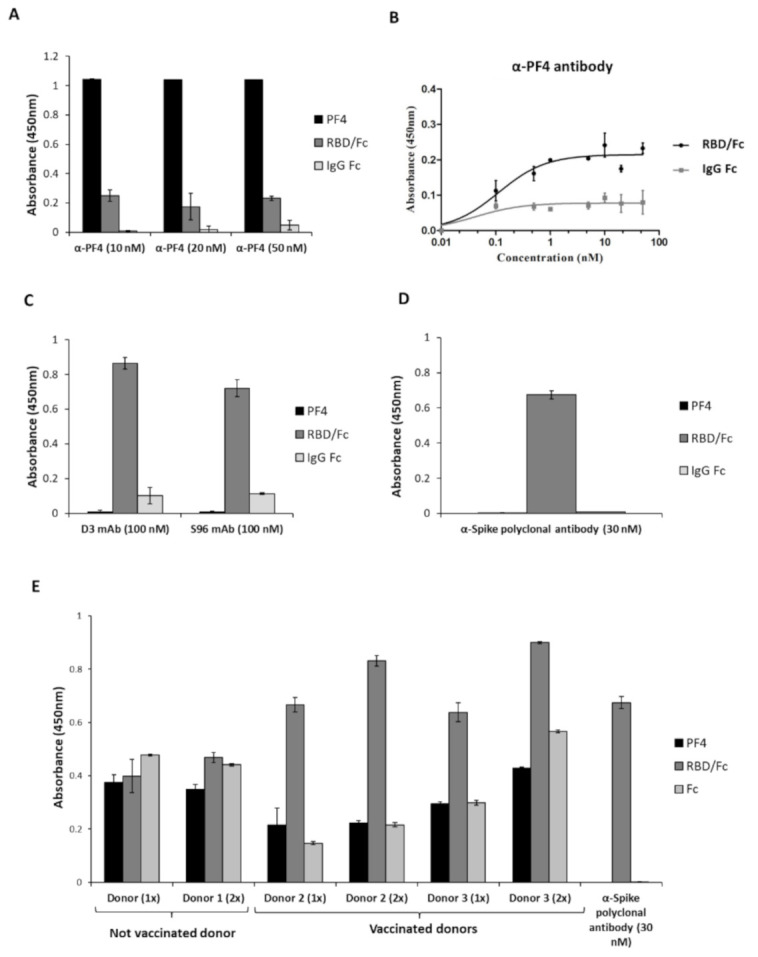
Cross-reactivity of the anti-PF4 or the anti-Spike-RBD antibodies for Spike-RBD and PF4, respectively. (**A**,**B**) Binding of anti-PF4 antibody to Spike-RBD of SARS-CoV-2. (**A**) ELISA assays were performed to test the binding of polyclonal anti-PF4 antibody to PF4 protein (black bars), to RBD/Fc chimeric protein (dark grey bars) or to Fc (light grey bars). (**B**) Binding curves of the polyclonal anti-PF4 antibody, tested at increasing concentrations on RBD/Fc (black curve) or Fc region (grey curve). Error bars depicted means ± SD. (**C**,**D**): Binding of the anti-Spike monoclonal and polyclonal antibodies to PF4. The novel human monoclonal D3 and S96 mAbs were tested by ELISA assays at the concentration of 100 nM on the immobilized PF4 protein (black bars), RBD/Fc protein (grey bars) or Fc region (white bars), used as positive or negative controls, respectively (**C**). The binding of the anti-Spike polyclonal antibody to the same proteins was tested at the concentration of 30 nM in a parallel assay (**D**). (**E**) ELISA assays to test the cross-reactivity for PF4 of the human anti-Spike polyclonal antibodies from vaccinated donors. The binding of aliquots of 15 μL (1×) or 30 μL (2×) of human plasma containing polyclonal antibodies of immunized donors was tested at increasing concentrations on RBD/Fc (grey bars) or PF4 (black bars) proteins immobilized on the plate at 5 μg/mL. The Fc domain (white bars) was used in parallel as a negative control. An anti-Spike polyclonal antibody was used as a positive control of specificity for RBD. Error bars depicted means ± SD.

Considering the similarity of the PF4 and RBD proteins, we investigated whether the antibodies specific for PF4 can recognize the Spike-RBD of SARS-CoV-2. To this aim, we performed ELISA assays by testing a polyclonal anti-PF4 antibody at the concentrations of 10, 20 and 50 nM on immobilized PF4, RBD/Fc or Fc protein, used in parallel assays as a negative control. As shown in Figure 2, the polyclonal anti-PF4 antibody recognizes the RBD even though the signal intensity was much lower than that observed on its specific PF4 target. To measure the binding affinity for RBD by the polyclonal anti-PF4 Ab, a dose response binding curve (0.1–50 nM) was performed on the immobilized Spike-RBD/Fc chimeric protein (Figure 2B) and on the Fc domain, used in parallel as a negative control. As shown in Figure 2B, a significant signal reaching saturation at the low concentration of 1 nM was observed when the anti-PF4 antibody was tested on RBD.

To evaluate the binding of the anti-Spike-RBD antibodies to the PF4 protein, we tested either human monoclonal (Figure 2C) or polyclonal (Figure 2D) anti-RBD antibodies by ELISA assays on the immobilized PF4 protein. As shown in Figure 2, both the monoclonal and polyclonal anti-RBD antibodies do not significantly bind to the PF4 protein, even at high concentrations of 30 or 100 nM.

To further confirm these data, we also tested the cross-reactivity for PF4 of plasma samples obtained from vaccinated donors containing high levels of human anti-Spike antibodies by ELISA assays. As shown in Figure 2E, the anti-RBD antibodies, produced by the vaccine injection, did not show significant binding to PF4 (black bars), as the signal intensity was comparable to that observed for the negative Fc control (white bars).

These findings suggest that the anti-RBD antibodies induced by vaccination do not recognize PF4, thus they are not responsible for platelet aggregation through PF4 binding; on the contrary they could prevent the unwanted binding of anti-PF4 antibodies to Spike-RBD.

**Figure 3:**
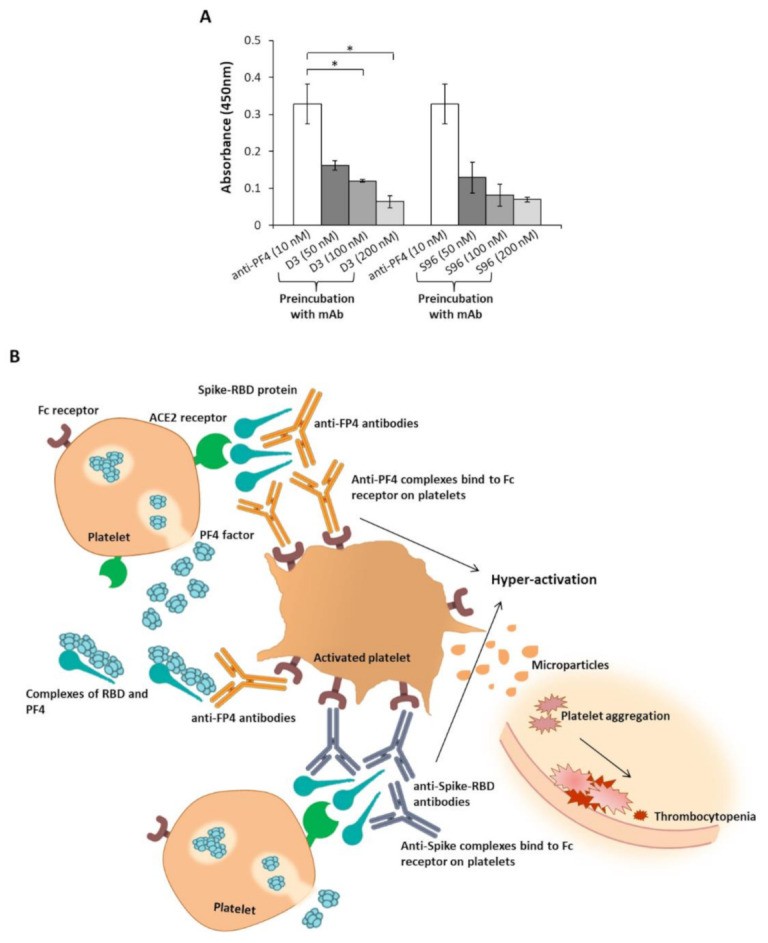
Interference of the novel anti-Spike mAbs in the interaction of the anti-PF4 antibodies with the Spike-RBD protein. (**A**) The binding to RBD was analyzed by testing the anti-PF4 antibody (white bars) in the absence or in the presence of D3 or S96 mAbs (grey bars) used at increasing concentrations. The binding of the anti-PF4 antibody was detected by using the anti-rabbit-HRP secondary antibody. Error bars depict means ± SD * *p* < 0.05. (**B**) A model explaining the possible roles of RBD and anti-PF4 antibodies in the platelets aggregation. Spike-RBD protein could bind to ACE2 receptor expressed on platelets. The recognition of Spike-RBD protein by either anti-PF4 or anti-Spike antibodies and the parallel engagement of the Fc receptor on activated platelets could induce the release of PF4 and formation of immune complexes with consequent platelets aggregation and thrombotic events.

To test this hypothesis, we performed competitive ELISA assays by using the novel neutralizing human anti-Spike D3 and S96 mAbs [19] to verify their ability to interfere in the interaction of anti-PF4 antibodies with RBD. To this aim, the polyclonal anti-PF4 Ab was incubated with the Spike-RBD protein in the absence or in the presence of a molar excess of D3 or S96 mAbs (used at increasing concentrations). Both the mAbs were found able to specifically interfere in the recognition of the RBD by the anti-PF4 antibody in a dose-dependent manner (Figure 3A).

Thus, considering that the novel human isolated anti-Spike mAbs can interfere in the recognition of RBD by the anti-PF4 antibody, they could be used to prevent the unwanted interactions of anti-PF4 mAbs to Spike that could contribute to platelets aggregation processes [6], such as those represented in Figure 3B, in particular if they are devoid of Fc fragment recognized by the Fc receptor on platelets.

### 2.3. Interaction of PF4 and Spike-RBD

**Figure 4:**
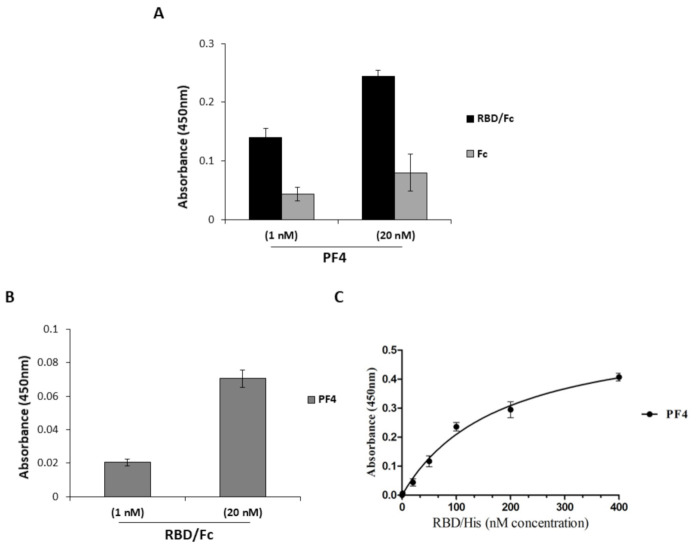
Binding between Spike-RBD and PF4. To test the ability of PF4 to bind to Spike-RBD protein, ELISA assays were performed by immobilizing either recombinant RBD/Fc protein or PF4 (5 μg/mL) and testing PF4 (**A**) or Spike-RBD protein (**B**), respectively, at increasing concentrations. The signal was detected by using the secondary rabbit anti-PF4 polyclonal antibody or the anti-Fc-HRP conjugated Ab, respectively. The Fc was used in a parallel assay as a negative control. Binding curve was obtained by incubating RBD/His protein at increasing concentrations on immobilized PF4 (**C**). The error bars depicted means ± SD.

To verify whether anti-PF4 antibodies could be generated by an interaction between PF4 and RBD proteins exposing new epitopes in a similar fashion to heparin-PF4 complexes [6,7,8,9], we tested the binding of the two proteins. To this aim, PF4 or RBD/Fc recombinant proteins were used at the concentrations 1 nM and 20 nM to test their respective binding to the immobilized Spike-RBD (Figure 4A) or PF4 (Figure 4B), respectively. The Fc was used in parallel assays as a negative control. As shown in Figure 4, in both cases a significant interaction between the Spike-RBD protein and the PF4 factor was observed. To measure the binding affinity of Spike-RBD to PF4, a dose response binding curve of RBD/His was performed on the immobilized PF4, and as reported in Figure 4C, a significant binding between the two proteins was confirmed with an apparent Kd value of 180 nM.

## 3. Discussion

A severe but rare complication of vaccination against SARS-CoV-2 is represented by platelets activation and subsequent coagulation abnormalities [1,2,3,6,7,8,9]. Even though the prevalence of these adverse events is very low, several countries instituted limits, based on age and sex, for the administration of vaccines based on adenoviral vectors, the main cause of VITT [6,8]. Most of the vaccinated patients who experienced these thrombotic events were women under 50 years of age undergoing hormone therapy. Recent evidence reporting the expression of ACE2 on platelet surface [8] suggest that Spike-RBD could bind to this receptor inducing platelets activation and the following release of cytokines and chemokines, such as PF4 [10,11]. Since in the plasma of vaccinated patients high levels of anti-PF4 antibodies have been recently detected, we firstly analyzed the structural similarities between Spike-RBD and PF4 and then investigated the eventual cross-reactivity of each specific antibody population for the other protein. In this study we evidenced the ability of polyclonal anti-PF4 antibodies to recognize the Spike-RBD, highlighting a possible direct association of Spike to platelets misregulation. These results are in contrast with those previously reported [20], showing a lack of cross-reactivity of affinity-purified anti-PF4 antibodies isolated from 14 VITT patients for SARS-CoV-2 spike protein, however we cannot exclude (as stated as well by the authors of that study) that eventual antibodies involved in VITT were bound in complexes and thus they were not accessible for purification or not able to bind to other molecules. We used instead a purified untreated polyclonal anti-PF4 antibody free to bind to all ligands and not deprived of eventual populations trapped in complexes with platelets or PF4.

Furthermore, since RBD and PF4 are able to interact with a significant affinity we formulate the hypothesis that they can form complexes in a similar fashion to heparin-PF4, that could be recognized by either anti-Spike or anti-PF4 antibodies. The recognition and the parallel engagement of the Fc receptor on activated platelets could induce the consequent platelets aggregation and thrombotic events, reported in vaccinated people who experienced VITT. These findings are in line with the previous reports showing the ability of Spike to induce platelets aggregation and ATP release in the presence of agonists in vitro and thrombosis in vivo on hAce2 transgenic mice [6]. In conclusion, we can assume a direct involvement of Spike protein, as well as PF4 chemokine and its specific antibodies in the onset of thrombosis and thrombocytopenia that should be investigated as well in in vivo studies. Furthermore, our findings indicate that diagnostic tests to detect the presence of anti-PF4 antibodies cross-reactive for Spike in the plasma before vaccination could be useful to avoid unwanted side effects. Further studies should be performed in the future to identify the sequences of Spike-RBD recognized by PF4 or anti-PF4 antibodies in order to eventually insert mutations in the future Spike-based vaccines or design peptides useful for interfering in these interactions.

## 4. Materials and Methods

### 4.1. Antibodies and Human Recombinant Proteins

The following human recombinant proteins and antibodies were used: human SARS-CoV-2 (2019-nCoV) chimeric Spike RBD-Fc protein (Sino Biological, 10108-H08H, Eschborn, Germany); human recombinant IgG1 Fc protein (R & D Systems, 110-HG, Minneapolis, MN, USA); human Platelet Factor 4 (Creative Biomart, PF4-52H, Shirley, NY, USA).

HRP conjugated anti-human Fc antibody (Sigma, AP113P, St. Louis, MO, USA); anti-human IgG (Fab’)2 goat monoclonal antibody (Abcam, ab98535, Cambridge, UK); rabbit polyclonal anti-PF4 antibody (Prodotti Gianni, ab9561, Milano, Italy).

### 4.2. Identification of Homologies between Human PF4 and SARS-CoV-2 Spike-RBD Protein and Comparison of Their 3D Structures

The following protein sequences were obtained from UniProtKB/Swiss-Prot portal: Spike-RBD protein (1273 aa)-P0DTC2; PF4 (101 aa)-P02776. The 3D structure of Spike-RBD and PF4 proteins were recovered from the UniProt 3D structure PBD database (https://www.ebi.ac.uk/interpro/protein/UniProt/P0DTC2/structure/PDB/#table and https://www.ebi.ac.uk/interpro/protein/UniProt/P02776/structure/PDB/#table (access date: 22 June 2021). PF4 and Spike-RBD were compared by referring to the published structures of the proteins [1,15,16].

### 4.3. ELISA Assays

To verify the binding of the anti-PF4 polyclonal antibody to Spike-RBD, ELISA assays were performed by testing the rabbit anti-PF4 polyclonal antibody (0.1–50 nM) on the immobilized chimeric Spike RBD-Fc protein, PF4 or Fc used in parallel as controls. The assays were performed as previously reported [19,21,22,23,24]. Briefly, NuncTM flat bottom 96-well plates (Thermo Fisher Scientific 439454 Ferentino, Italy) were coated with 5 μg/mL Spike RBD-Fc, PF4 or Fc recombinant proteins in a solution of 0.05 M NaHCO3 for 72 h at 4 °C. After blocking with 5% nonfat dry milk in PBS for 1 h at 37 °C, the purified Abs were added at increasing concentrations to the plates in 3% Bovine Serum Albumin (BSA Sigma A7030, St Louise, MO, USA) in PBS and incubated for 90 min by gently shaking. After the first incubation, extensive washes were carried out with PBS, and the plates were incubated for 1 h with the secondary HRP-conjugated antibody in PBS containing 3% BSA. After washes the plates were incubated with a 3,3′,5,5′-tetramethylbenzidine (TMB Sigma-Aldrich T0440, St Louise, MO, USA) reagent. Absorbance at 450 nm was measured by the Envision plate reader (Perkin Elmer 2102, Milano, Italy).

To test the binding of the novel human monoclonal or polyclonal anti-Spike mAbs to the immobilized PF4 or to RBD/Fc proteins, ELISA assays were performed by testing increasing concentrations of D3, S96 mAbs or the anti-Spike polyclonal Ab. Similarly, aliquots (15 or 30 μL) of human plasma from vaccinated donors were tested in the same conditions [19,21,22,23,24].

In order to investigate the ability of the novel isolated D3 and S96 mAbs to interfere in the binding of the anti-PF4 Ab to the Spike-RBD protein, competitive ELISA assays were performed by measuring the binding of the anti-PF4 Ab in the absence or in the presence of a molar excess of D3 or S96 mAbs (5:1 or 20:1 M/M). The assays were performed as previously described [19,21].

Binding values were reported as the mean of at least 3 determinations obtained in 3 independent experiments^.^ The Kd value was calculated by the analysis of binding curves with the Graphpad Prism software, as previously reported [24]. The equation model used was: Y = Bmax × X/(Kd + X) + NS × X + Background. Bmax is the maximum specific binding in the same units as Y; Kd is the equilibrium binding constant, in the same units as X, and it is the ligand concentration needed to achieve a half-maximum binding at equilibrium; NS is the slope of non-specific binding in Y units divided by X units; background is the amount of nonspecific binding with no added ligand.

### 4.4. Statistical Analysis

Error bars were calculated on the basis of the results obtained by at least 3 independent experiments and represent means ± SD. For the competitive ELISA assays the two-tailed Student’s t-test was applied and the statistical significance was defined as *** *p* ≤ 0.001; ** *p* < 0.01; * *p* < 0.05.

## Data Availability

The study did not report any data.
